# New Local Anæsthetic

**Published:** 1874-05

**Authors:** 


The New Local Anæsthetic.—The observation of Horwath, of Kiew, that absolute alcohol at a temperature of twenty degrees Fahr. is a most efficient local anæsthetic, deserves to be remembered and acted upon. He finds it far superior to cold ether, or ice, or the spray of volatile substances.

